# Deep Learning-Based Anomaly Detection in Video Surveillance: A Survey

**DOI:** 10.3390/s23115024

**Published:** 2023-05-24

**Authors:** Huu-Thanh Duong, Viet-Tuan Le, Vinh Truong Hoang

**Affiliations:** Faculty of Information Technology, Ho Chi Minh City Open University, 97 Vo Van Tan, District 3, Ho Chi Minh City 700000, Vietnam; thanh.dh@ou.edu.vn (H.-T.D.); tuan.lv@ou.edu.vn (V.-T.L.)

**Keywords:** abnormal human activity recognition, deep learning, video surveillance, anomaly detection

## Abstract

Anomaly detection in video surveillance is a highly developed subject that is attracting increased attention from the research community. There is great demand for intelligent systems with the capacity to automatically detect anomalous events in streaming videos. Due to this, a wide variety of approaches have been proposed to build an effective model that would ensure public security. There has been a variety of surveys of anomaly detection, such as of network anomaly detection, financial fraud detection, human behavioral analysis, and many more. Deep learning has been successfully applied to many aspects of computer vision. In particular, the strong growth of generative models means that these are the main techniques used in the proposed methods. This paper aims to provide a comprehensive review of the deep learning-based techniques used in the field of video anomaly detection. Specifically, deep learning-based approaches have been categorized into different methods by their objectives and learning metrics. Additionally, preprocessing and feature engineering techniques are discussed thoroughly for the vision-based domain. This paper also describes the benchmark databases used in training and detecting abnormal human behavior. Finally, the common challenges in video surveillance are discussed, to offer some possible solutions and directions for future research.

## 1. Introduction

With today’s increasing demand for security, especially in public places such as airports, train stations, supermarkets, schools, and crowded street, surveillance cameras are used for monitoring daily activities and detecting abnormal events. This task focuses on the localization of anomalies using both temporal and partial information in videos. Anomalies can be defined as events deviating from normal behavior [1], e.g., fighting, sneaking, or unattended bags at an airport. The purpose of using surveillance cameras is the early detection of anomalous human behaviors. This is a critical task in many cases where human intervention is necessary, e.g., for crime prevention or countering terrorism. However, this process requires labor-intensive and continuous human attention, which is a tedious process, since abnormal events only happen 0.01% of the time and 99.9% of the surveillance time is wasted [2]. Moreover, a surveillance system produces a lot of redundant video data, which require unnecessary storage space. For reducing human errors and storage costs, it is necessary to build an efficient surveillance system for detecting any strange behaviors that may lead to dangerous situations. This requires deep and comprehensive study of human activity recognition, to understand the features representative of each action.

Anomaly detection in video has a wide range of applications, such as for traffic accident detection, criminal activity detection, and illegal activity detection. In addition, detecting anomalous items or abandoned objects, such as guns or knifes, is necessary in sensitive areas.

Anomaly detection has become an active area of research in recent years. With the purpose of trying to conduct an automated process for detecting abnormal events, many methods have been proposed. The essential idea is to first learn normal patterns from training videos, then extract representations of normal cases. If any events deviate from these representations, an abnormal event can be detected. However, there are still a lot challenges in video surveillance and human activity recognition. These difficulties are related to the feature extraction stage, where occlusion, overlapping, cluttered backgrounds, sensor noise, low illumination, and dynamic background changes may impact a system’s performance [3]. It is quite challenging to extract robust and discriminative features for training models due to these various challenges. Moreover, anomaly detection is also dependent on the scene context [4], where one action is considered abnormal in one scene but may be normal in another. This requires a vast amount of data for training, to verify all cases happening in the real world.

This survey explores the current deep learning methods that have been used in video surveillance. In particular, it focuses on the understanding of abnormal human behaviors, since the identification and recognition of actions is key for an intelligent video system. This survey’s scope encompasses vision-based approaches, since this is becoming a popular trend in action recognition. The significant contributions of this survey are as follows:

- A comprehensive review of vision-based human activity recognition for video surveillance.
- A description of popular databases used in anomaly detection.
- Analysis of data processing and feature engineering for deep learning models.
- Discussion of recent deep learning models, along with their advantages and disadvantages.
- Identifying existing challenges and future research for anomaly detection in video surveillance.

In order to prepare for the survey, an essential step was to search and filter recent papers on video surveillance using deep learning techniques for understanding human activities. The research papers were download using relevant index terms, such as *video surveillance, deep learning activity recognition, human action representation, abnormal behavior detection.* The references from these papers were extracted, validated, and added to the survey. After that, research relevant to video surveillance was also included. Finally, the general structure and representative methods were provided in detail, to give an insight into each learning model.

The organization of this survey is as follows: In Section 2, the background and the related surveys are provided. A discussion of common databases used in video surveillance is provided in Section 3. Section 4 explains data processing and feature engineering in the vision-based domain. A detailed analysis of the state-of-the-art deep learning models is provided in Section 5. Section 6 discusses the existing challenges in anomaly detection and gives directions for future research. Finally, Section 7 concludes the survey.

## 2. Background Knowledge and Related Works

### 2.1. Background Knowledge

Human action recognition (HAR) is a fundamental problem in computer vision and has been studied for a long time. The purpose of HAR is to identify the action taking place in a video, in order to understand and produce an analysis of specific events. A video’s spatial and temporal information plays an important role in HAR, to correctly identify human actions and classify the video. The detection of abnormal behavior in video surveillance is basically used to ensure security in both indoor and outdoor locations, such as in airports and train stations. Abnormal human action recognition (AbHAR) can be considered a particular problem in HAR. The problem with AbHAR in videos is that it can vary widely, and there is no single approach to solving all problem cases. The common approaches to AbHAR rely on feature extraction from image sequences. These features are built for the tasks of object detection, pose estimation, and finding the dense trajectories that are useful in HAR.

AbHAR is used in video surveillance for monitoring behavior and people’s activities, with the purpose of ensuring security and giving instructions. This requires a certain general background in many video processing domains. Video surveillance includes the implementation of knowledge of feature extraction, scene understanding, object tracking, object identification, and model generation. Specifically, the feature extraction step needs to be addressed carefully, since it has a great impact on the whole system. Traditional machine learning algorithms have achieved remarkable results in AbHAR, based on learning shallow features from video data. Methods such as random forest (RF) [5], Bayesian networks [6], Markov models [7], and support vector machine (SVM) [8] have been used to understand and recognize human behavior. These methods are heavily dependent on preprocessing and handcrafted features, which require a lot of time and resources to process. Furthermore, they do not scale well for different datasets and show a poor performance with real cases [9]. In recent years, deep learning methods have achieved great interest from the research community, since they can automatically extract learning features and have shown promising results for difficult research topics, such as object detection and recognition [10], image classification [11], and natural language processing (NLP) [12]. Compared to traditional ML methods, deep learning is a multistage learning process that automatically extracts the representative features for a specific task utilizing several hidden layers [13]. These features are called deep features, and they can be scaled reasonably well to various scenarios. Deep learning has recently been applied to HAR and AbHAR, and has proven to be very efficient for video surveillance systems [14].

**Figure 1:**
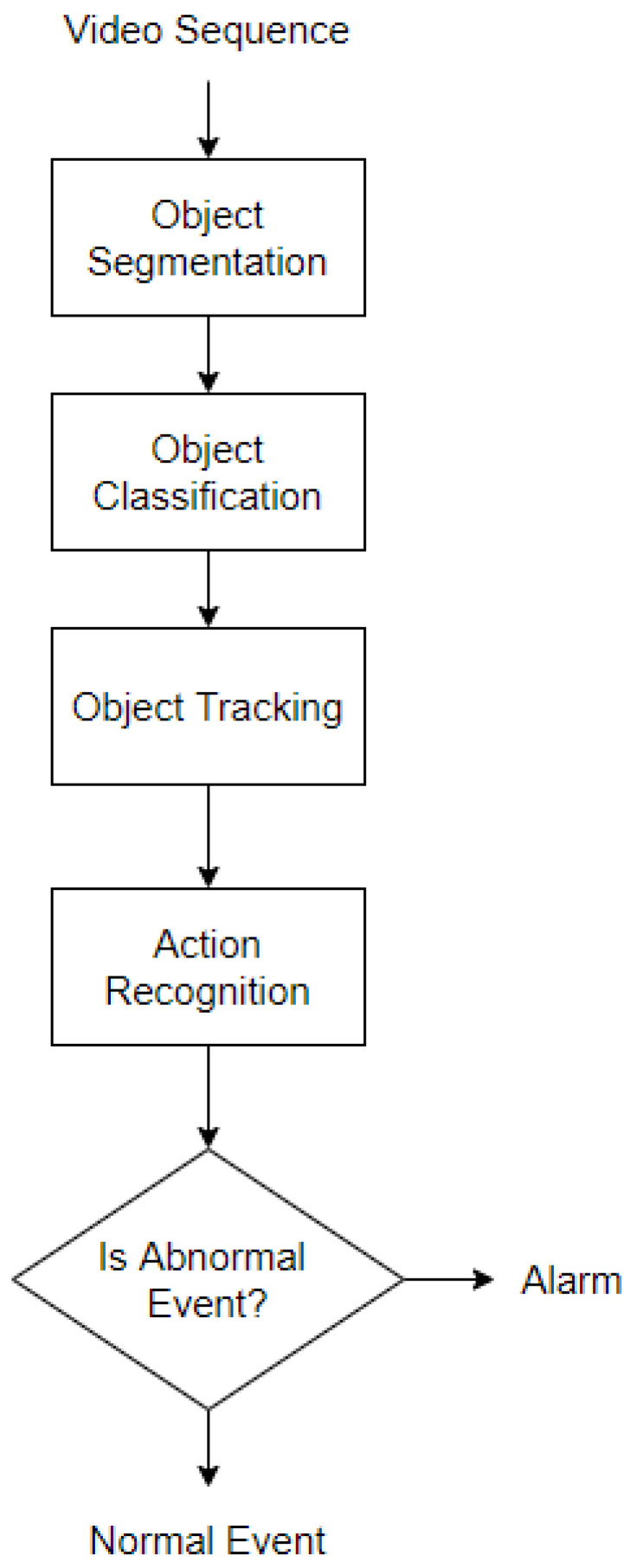
General Process of Video Surveillance.

Figure 1 shows a the diagram of the primary processing steps for AbHAR in video surveillance. In general, there are four main steps: object segmentation, object classification, object tracking, and action recognition. Object segmentation localizes moving objects (mainly human targets) on the scene. Object classification identifies the type of each object of interest for later processing. These two steps are heavily reliant on extracting features from video data. Then the targets of interest are tracked through the frame sequence using object tracking algorithms. Object tracking methods refer to motion estimation and re-identification for the tracking of targets. Lastly, action recognition learns representative features to classify and understand which type of anomalous activities represent each tracking object. If abnormal behavior is detected, then an alarm or notification is sent to the authorities.

### 2.2. Related Works

Anomaly detection in video surveillance has been studied for decades. The topic has attracted much attention from the research community, due to its varied applications. A vast amount of methods have been proposed for both traditional and deep learning approaches to AbHAR. In order to keep track of all available studies, thorough surveys are of considerable interest. In 1997 and 1999, there were reviews [15,16] on human motion analysis applied in HAR. In 2005, automated video surveillance systems were surveyed in [17]. Three years later, a study of behavior analysis for homeland security applications [18] was published. In 2012, the authors in [19] provided a survey of anomaly detection in video surveillance applications, within different contexts.

In 2013, a survey was performed of vision-based HAR [20], which reviewed the existing research on HAR. The survey focused on the use of the self-evaluation method to extract important features and also suggested some directions for future research. In 2014, the authors in [21] summarized the different techniques used for intelligent surveillance systems in public places. Another review of crowded scene analysis [22] was conducted in 2014. In the subsequent two years, the authors in [23] focused on various HAR methods for video streams. One year later, there was another comprehensive review [24] covering both handcrafted and deep feature representations. At the same time, behavior representation and behavior modeling were both discussed in [25].

A survey [26] in 2018 covered the use of RGB-D-based methods for action detection and classification. The authors also provided a detailed discussion of the strengths and weakness of each anomaly detection method. In the same year, the relationship between HAR and data stream mining was thoroughly summarized in [27]. There was also a discussion of unsupervised and semi-supervised anomaly detection in [28]. In 2019, there were a lot of surveys of HAR. The authors of [29] performed a comprehensive review focused on the three different aspects of feature presentation, interaction recognition, and human action detection methods. The authors of [30] reported different processing techniques for video surveillance. The review in [31] focused on both handcrafted and deep approaches for different types of two-dimensional and three-dimensional data. Most recently, the survey in [32] detailed various techniques for singe-scene video anomaly detection. The authors in [33] provided a comprehensive survey that discussed both sensor-based and vision-based human activity recognition.

A survey of network anomaly detection [34] was performed by Ahmed et al. They focused on four types of network attack; namely, denial of service, probe, user to root, and remote to user. They grouped the research methods into four categories, including classification-based network anomaly detection, statistical anomaly detection, information theory, and clustering-based. They also discussed some intrusion detection datasets. Another survey [35] reviewed anomaly based network intrusion detection systems (NIDSs), which is an important field in cybersecurity. The authors grouped methods into six categories, as in [34], and provided further details about benchmark datasets. A more detailed survey [36] on network anomaly detection was published in 2019. This survey was split into two main parts. The first part discussed anomaly detection background analysis, considering traffic anomalies, network data types, and intrusion detection systems. Network traffic anomalies were divided into two categories: anomaly categorization based on its nature, and based on its causal aspect. They also discussed two types of intrusion detection systems (IDS), which are IDS types using a monitored platform and IDS types using a detection technique. The second part mainly discussed anomaly detection methods, techniques, and systems. This part included statistical, clustering, finite state machine, classification, information theory, evolutionary computation, and hybrid/other methods.

## 3. Benchmark Databases

Anomaly detection has been extensively studied in many fields, especially in computer vision, for the purpose of learning and understanding activity recognition. This task can become very challenging, due to the complexity of real-world situations. There are infinite abnormal events, and it is impossible to gather all kinds. Fortunately, many databases have been developed to help the scientist and researcher with this task. These databases were mainly acquired in the visible spectrum, so they are sensitive to occlusion and illumination changes. In this section, we briefly introduce some popular databases that researchers are currently using for detecting behavioral anomalies. We arrange the databases in chronological order, from the oldest to the newest database. For each database, we summarize information of the release year, type of database (i.e., single-scene or multiscene), details of the sensor used (RGB or thermal, resolution, FPS), description of anomalous activities, and sample images.

### 3.1. CASIA Action Database

**Figure 2:**
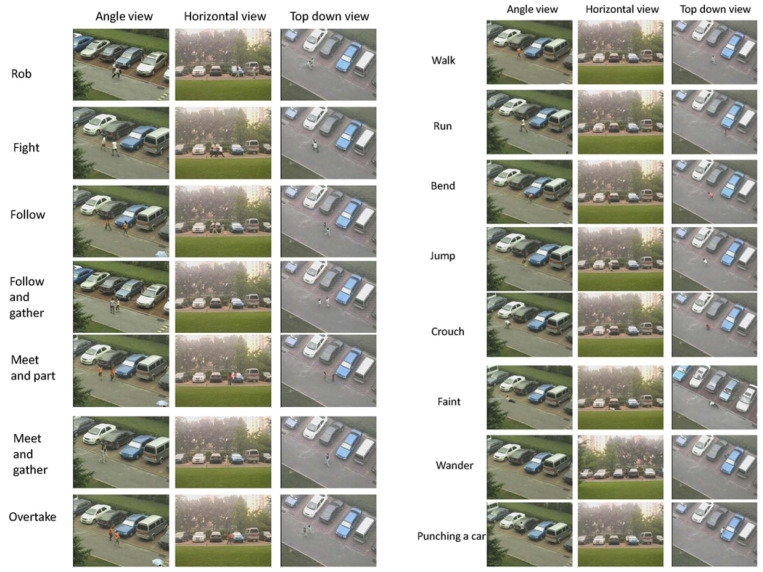
Sample images from the CASIA database. From left to right: single person action, two person interaction [37].

The CASIA Action database [37] was released in 2007 and is a type of RGB single-scene dataset, i.e., it was recorded with color sensors in a certain location. This database includes human outdoor activities captured from different viewing angles. There are eight types of action for a single person, such as walk, run, bend, jump, crouch, faint, wander, and punching a car, from 24 different subjects. There are also seven types of two person interaction, such as rob, fight, follow, follow and gather, meet and part, meet and gather, overtake. The database includes two main parts: single person actions, and interactions (see Figure 2). A description is given below:

- Single person action:
  - Walk: One subject walking along the road.
  - Run: One subject running along the road.
  - Bend: One subject bending his/her body while walking.
  - Jump: One subject jumping along the road.
  - Crouch: One subject crouching once while walking along the road.
  - Faint: One subject falling down on the ground while walking along the road.
  - Wander: One subject wandering around.
  - Punching a car: One subject punching a car.
- Interaction:
  - Rob: One subject robing another one.
  - Fight: Two subjects fighting with each other.
  - Follow: One subject following another till the end.
  - Follow and gather: One subject following another and then walking together with the other to the end.
  - Meet and part: Two subjects meeting each other and then departing.
  - Meet and gather: Two subjects meeting each other and then walking together till the end.
  - Overtake: One subject overtaking another.

In order to capture the videos, three noncalibrated cameras were used simultaneously from different viewing angles, which were a horizontal view, angled view, and top-down view. These videos were encoded at a frame rate of 25 FPS and were compressed with the huffyuv codec in avi format. The original spatial resolution of the video frames was reduced to 320 × 240. Each video lasts from 5 s to 30 s, due to the different kind of action. Figure 2 shows some images from the database.

### 3.2. Subway Database

**Figure 3:**
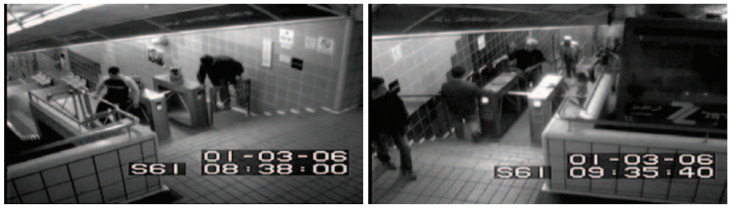
Sample images from the Subway Database. From left to right: subway entrance, subway exit [38].

The subway database was introduced by Adam et al. in their 2008 paper [38]. This is also a type of single-scene database. This database comprises two long video recordings monitoring people at a subway entrance and exit, see Figure 3. There is no spatial ground truth available. The video was recorded in grayscale format at 15 FPS with a resolution of 512 × 384, and it has 125,475 frames in total. The anomalous events are mainly wrong directions, loitering, no payment, people jumping or squeezing through turnstiles, and a janitor cleaning the walls.

### 3.3. UMN Crowd Abnormality Database

The UMN database was released in 2009 and first described in the paper titled “Abnormal crowd behavior detection using social force model” by R. Mehran et al. [39]. The video scenario simulated a populated area, where actors are wandering around a certain location and run away in escape mode with abnormal behavior. Therefore, this database can be considered a multiscene database, meaning that it was recorded in different locations.

**Figure 4:**
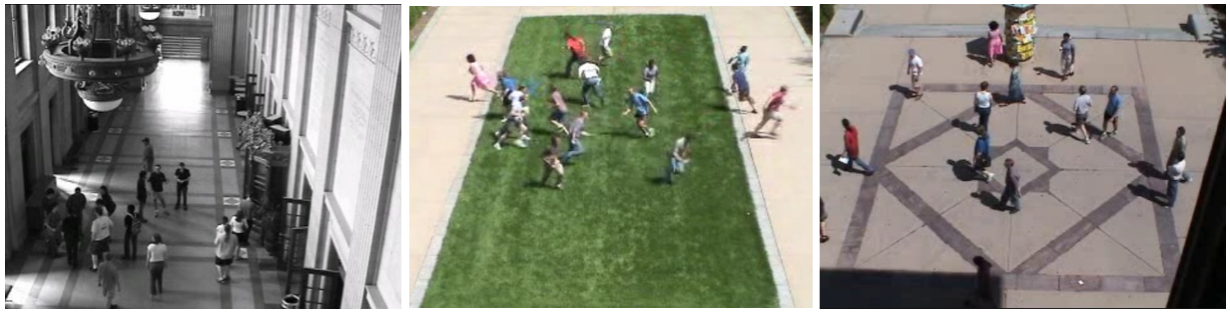
Sample images from the UMN database [39].

The database has a total of 11 short videos, which are aggregated into one long video of 4 min 17 s with 7739 frames. The small videos begin with normal behavior, then change to abnormal. There is one scenario for the indoor scene and two outdoors. All the videos have the same frame rate of 30 FPS and were recorded at a resolution of 640 × 480 using a static camera. The ground truth is a temporal annotation. Figure 4 provides some sample images from the video.

### 3.4. Anomalous Behavior Database

**Figure 5:**
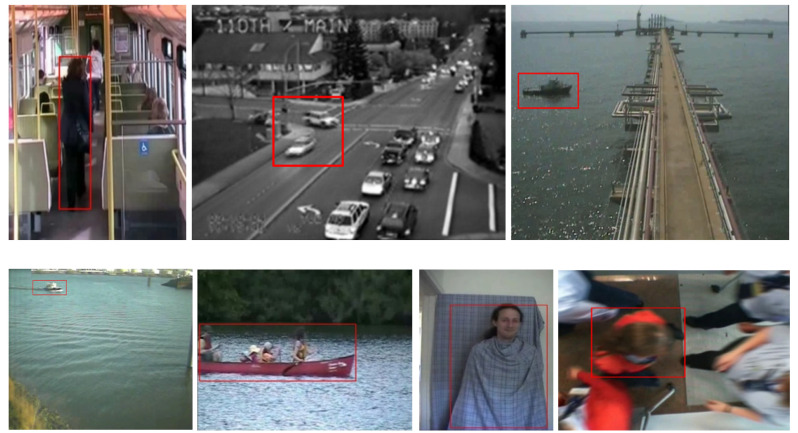
Sample images from the Anomalous Behavior Database. From left to right, and top to bottom: Traffic–Train, Traffic–Belleview, Boat–Sea, Boat–River, Canoe, Camouflage, Airport–WrongDir [40].

The Anomalous Behavior Database [40] was released in 2010 by York University. The database comprises eight videos (multiscene) recorded in various challenging conditions, such as illumination effects, scene clutter, variable target appearance, rapid motion, and camera jitter. The database was also provided with a spatiotemporal ground truth, along with software for detecting abnormal events in certain parts of each video. The image sequences in this database mainly focus on the activities of humans and vehicles in certain public locations, such as an airport, river, sea, and on a train, see Figure 5:

- Traffic–Train: This video records daily activity on a train. This is a very challenging video, since the lighting conditions change drastically and there is camera jitter. The video includes 19,218 frames of RGB image with a resolution of 288 × 386 and a frame rate of 25 FPS. The anomalous event is the movement of a passenger.
- Belleview: This video includes cars moving through an intersection. Video is recorded in grayscale format at a resolution of 320 × 240, with a frame rate of 10 FPS, and it has 2918 frames in total. The anomalous event is cars entering the thoroughfare from the left or right.
- Boat–Sea: This video describes a passing boat as an abnormal event. The video was recorded in RGB format at a resolution of 720 × 576, with a frame rate of 19 FPS, and it has 450 frames in total.
- Boat–River: This video illustrates a boat passing on a river as an abnormal event. The video was recorded in RGB format at a resolution of 720 × 576, with a frame rate of 5 FPS, and it has 250 frames in total.
- Canoe: This video describes a canoe passing on a river as an abnormal event. The video was recorded in RGB format at a resolution of 320 × 240, with a frame rate of 30 FPS, and it has 1050 frames in total.
- Camouflage: This video illustrates a person walking in camouflage. The right motion is learned as the normal behavior, and the opposite is the abnormal behavior. The video was recorded in RGB format at a resolution of 320 × 240, with a frame rate of 30 FPS, and it has 1629 frames in total.
- Airport-WrongDir: This video records people walking in a line at an airport. The video was recorded in RGB format at a resolution of 300 × 300, with a frame rate of 25 FPS, and it has 2200 frames in total. The anomalous event is people moving in the wrong direction.

### 3.5. Avenue Database

**Figure 6:**
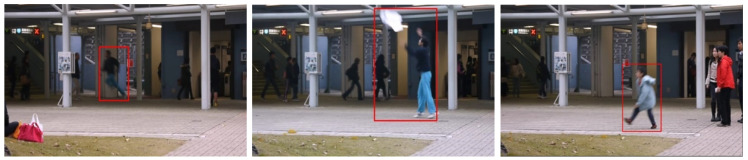
Sample images from the Avenue Database. From left to right: running, throwing object, and loitering [41].

This database [41] was released in 2013 and contains 37 videos, divided into 16 normal videos for training and 21 abnormal videos for testing. The database is of a RGB single-scene type. There is a total of 47 abnormal events, categorized into three main subjects: strange actions, wrong direction, and abnormal object. These videos were captured at CUHK campus avenue, with 30,652 frames (15,328 training, 15,324 testing) in total. Each image sequence has a resolution of 640 × 360 and frame rate of 25 FPS. The author provided both temporal and spatial annotations. Three main types of abnormal events (see Figure 6) are defined below:

- Strange actions: behaviors such as running, throwing objects, and loitering.
- Wrong direction: people moving in the wrong direction.
- Abnormal objects: people carrying some strange objects with them, such as a bicycle.

### 3.6. UCSD Anomaly Detection Database

This database [42] was released in 2013 and includes two subdatasets: Pedestrian 1, and Pedestrian 2. Both include a grayscale sequence of images record at 10 FPS with a resolution of 238 × 158 for pedestrian 1, and 360 × 240 for pedestrian 2. Each dataset is a single-scene dataset. Both have training videos containing only normal behaviors and testing videos containing abnormal events. A static camera was used to record the dataset and was setup on an elevator overlooking the pedestrian walkways. The abnormal events include the following:

- The circulation of non-pedestrian entities on the walkways such as bikers, skaters, and small carts.
- Anomalous pedestrian motion patterns, such as people walking across a walkway or in the grass that surrounds it.

**Figure 7:**
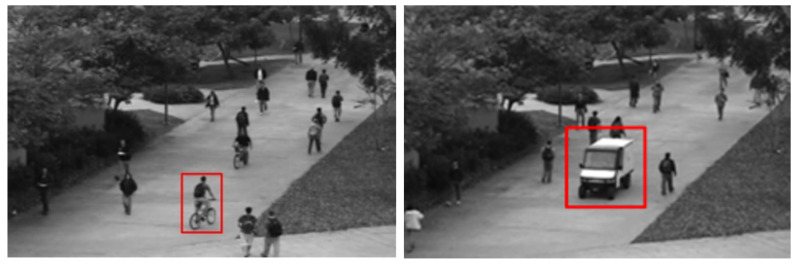
Ped1 subdataset with nonpedestrian entity [42].

The Pedestrian 1 (Ped1) dataset includes 34 normal training videos and 36 abnormal testing videos of groups of people walking towards and away from the camera. These abnormal cases are mainly related to abnormal vehicles, such as bicycles and cars entering the crowd. Some sample images are shown in Figure 7.

**Figure 8:**
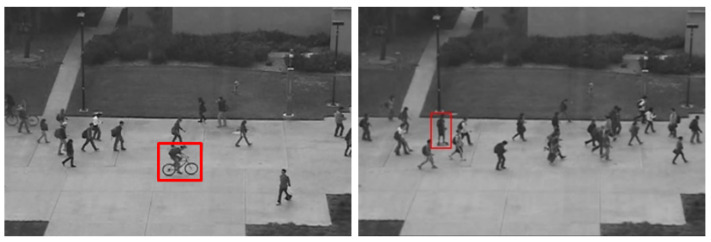
Ped2 subdataset with nonpedestrian entity [42].

The Pedestrian 2 (Ped2) dataset contains 16 training videos and 12 testing videos with 12 abnormal events. Ped2 includes scenes with pedestrian movement parallel to the camera plane. The definition of an anomaly for Ped2 is the same as for Ped1. Some example images from the Ped2 dataset are shown in Figure 8.

### 3.7. ShanghaiTech Campus Database

**Figure 9:**
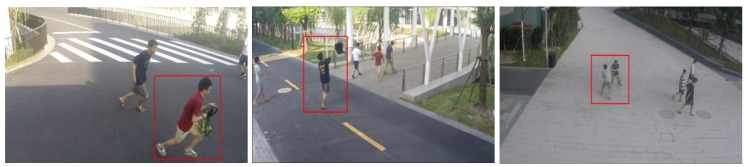
Sample images form the Avenue Database. From left to right: robbing, throwing object, and fighting [43].

This database [43] was release in 2016. It contains 330 training videos with only normal events, and 107 testing videos with 130 abnormal events. The total frames are 317,398, with 17,090 irregularity frames. The database was acquired using an RGB camera with a resolution of 856 × 480 at 24 FPS, overlooking pedestrian walkways. It consists of 13 scenes (multiscene) with complex light conditions, camera angles, and various anomaly types, mainly related to strange objects, wrong direction, and strange actions (see Figure 9).

- Strange actions: behaviors such as running, robbing, pushing, jumping, jumping over the fence, dropping, throwing objects, and fighting. Below are some sample images.
- Wrong direction: there is the case where people usually follow a normal direction, but someone does the inverse.
- Abnormal objects: this is a case where a person carries a strange object with them, such as a bicycle or baby stroller.

### 3.8. UCF-Crime Database

**Figure 10:**
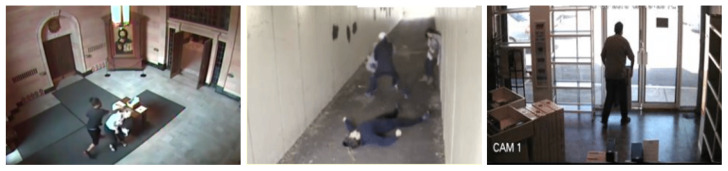
Sample images from the UCF-Crime Database. From left to right: abuse, fighting, and shoplifting [2].

The UCF-Crime database was released in 2018 [2]. This database is a compilation of 128 h of 1900 internet videos, taken from many RGB cameras at different locations (multiscene). The anomalous events include abuse, arrest, arson, assault, road accident, burglary, explosion, fighting, robbery, shooting, stealing, shoplifting, and vandalism. These videos cover 13 real-world situations and can be used for two primary tasks: the event recognition of 13 group activities, and anomaly detection in each specific group. The authors only provided temporal annotations. Figure 10 shows some sample images from the database.

### 3.9. Street Scene Database

**Figure 11:**
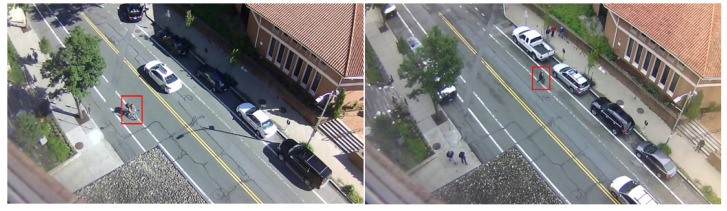
Sample images from the Street Scene Database. From left to right: biker outside lane and jaywalking [44].

The Street Scene database is the most recent database, being released in 2020 [44]. This dataset is a RGB single-scene type. There are a total of 203,257 image sequences, extracted from the original videos at a frame rate of 15 FPS. The database consists of 205 anomalous events, such as jaywalking, biker outside lane, and loitering… recorded at a resolution of 1280 × 720, see Figure 11. This database is challenging, due to the various activities, occlusions, shadows, and moving backgrounds.

## 4. Preprocessing Data for Video Anomaly Detection

The discussed benchmark databases are just collections of raw data of videos and images. These data need to be preprocessed before feeding into the ML algorithms. Data preparation and feature engineering are considered two important processes that greatly affect the performance of a video surveillance system. For the vision-based domain, these processes include several steps, such as background construction, foreground extraction, and feature representation extraction. The main purpose of the feature engineering process is reducing noise, selecting important representation features, transforming high-dimension features into the sub-space domain without losing valuable information, and reducing the overfitting problem. However, there are a lot of challenges, such as variation in lighting conditions, cluttered backgrounds, occlusion, or spurious interactions between subjects.

### 4.1. Segmentation

The first step in data preprocessing is segmentation. Segmentation is used to extract the target subjects from images or videos. Segmentation includes background construction and foreground extraction techniques. Background construction algorithms model background information by trying to determine a scene’s global representative features, and the identified subjects are then analyzed based on the difference between the current frame and the constructed background [45]. Background construction techniques are used for tracking of fast objects in a scene, and they are computer efficient when used with fixed-cameras. Some statistical methods of background construction, such as [46,47,48], can work well in the multi-modal domain with adaptive parameters; however, their performance is greatly reduced by environmental noise or poor lighting conditions. The neural network based-techniques reported in [45,49,50] can overcome these limitations, but they easily overfit data. The choice of technique mainly depends on the application’s purpose. For dynamic background recording with moving cameras, it is necessary to use a foreground extraction-based segmentation algorithm. Both spatial and temporal information are analyzed from a video sequence to extract target subjects from the background. The authors in [51,52] used an optical flow technique to deal with occlusion and distortion, and successfully extracted the targets from the video recording by moving the camera; however, their methods is complex and time-consuming. Temporal information was also used in [53,54] to perform foreground segmentation, which was sensitive to noise but required a low computing power. References [55,56] used Markov random fields to preserve the boundaries and handle complex backgrounds, but these methods are not computer efficient.

Many of the methods proposed for video anomaly detection have tried to exploit both background and foreground information. Lai et al. [57] introduced a network that included two decoders to generate a future frame and RGB difference. Doshi et al. [58] used a pretrained object detection model (YOLO) to capture location and appearance features by detecting objects in a video. On the other hand, Cai et al. [59] used an image clip and its optical flow clip as input to capture structure and motion information. Then, two decoders generated a future frame and optical flow image using the fused feature, which was extracted from two encoders.

### 4.2. Feature Extraction and Selection

Handcrafted-feature-based extraction [60,61] was used to extract useful features for understanding human behaviors. These methods are restricted to certain conditions, and lack the flexibility to adapt to new environments. They are also time consuming and computer inefficient. Researchers turned to new representative features, which are categorized into three different types: local, global, and semantic features. These features have clearly shown their advantages and robustness to noise and dynamic environments.

- Local representative features use local descriptor algorithms to govern how an input region of an image is locally quantified. They take into account the locality of regions in an image and describe them separately. A HOG (histogram of gradients), as reported in [60,62,63], is a basic technique to extract a local description of the gradient magnitude and orientation of images. A HOG is invariant to photometric transformations but can only be used for human detection at fixed size. Scale-invariant feature transform (SIFT) was used in [64,65] and showed invariance to geometric and photometric transformation, even with 3D projection, but it contains high-dimensional features, which is computer inefficient and unsuitable for real-time applications. The speed-up robust feature (SURF) algorithm [66,67] is an alternative to SIFT that is faster and retains the detection points’ quality. Lastly, the shape-based local feature descriptor in [68,69] demonstrated its robustness to noise by preserving the edge structures of the target subjects. However, it is heavily dependent on silhouette segmentation in its preprocessing step.
- Global representative features use an image descriptor that governs how an input image is globally quantified and returns a feature vector abstractly representing the image contents. The global descriptor in [31,70] encoded the detail information of corners, edges, ridges, and the optical flow as essential features. These features can be easily obtained from the camera depth but they are scene-dependent and have a lack of generic info. Some researchers in [71,72,73] used 3D space–time volume to extract 3D global feature vectors, which were independent of background subtraction. However, these 3D features were highly sensitive to noise and occlusion. The discrete Fourier transform (DFT) was also used in [74,75] to transform spatial features into frequency features, but the inverse process may lose the spatial and temporal information needed to identify the anomalous target subjects.
- The semantic features are obtained from the analysis of human body postures in a video sequence. These features can be transformed into human pose information [76,77] that is robust to interclass variation, but pose accuracy is hard to achieve. The researchers in [78,79] used appearance-based features such as textures and colors to gain contextual information, but this was sensitive to intraclass variations. Three-dimensional semantic features can be obtained from RGD-D cameras [68,80], which provide both geometric and visual information, but this is highly affected by noise and occlusion problems.

## 5. Deep Learning Methods

This section discusses the deep learning methods used in AbHAR in video surveillance. Deep learning methods have achieved significant results, in term of both behavior recognition and video comprehension. A deep learning system can automatically learn and extract representative features from image sequences that contain both spatial and temporal information, using a AbHAR process. Based on different strategies, processing techniques, and the final objective of the network architecture, these deep learning methods can be categorized into four main groups; namely, reconstruction-based methods, multiclass classification methods, future frame prediction methods, and scoring methods. Table 1 summarizes the deep learning-based techniques used for anomaly detection in different categories.

### 5.1. Reconstruction-Based Method

This is the most common method used in AbHAR [82,92]. Formally, let *x* be the input image sequences and *f* be the neural network that reconstructs *x*. The reconstruction cost function Θ can be defined as a function to compute the error *e* between the original input *x* and f(x), see Equation (1).

(1) e=Θ(x,f(x))

**Figure 12:**
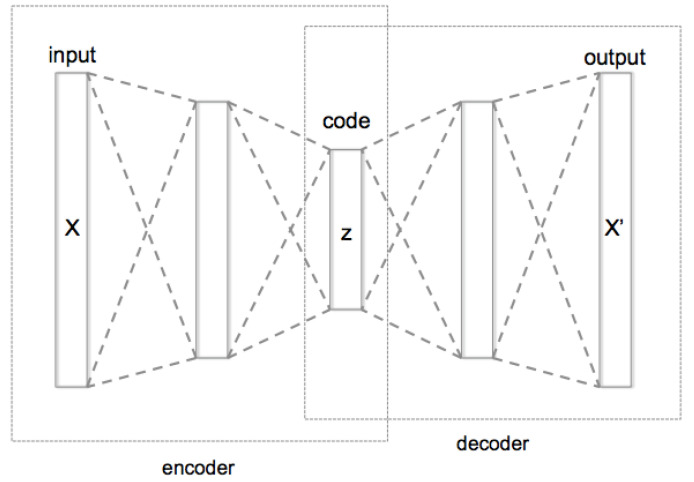
Autoencoder schematic structure [98].

A popular neural network used for the reconstruction error function Θ is an autoencoder network. An autoencoder network has two parts: an encoder and decoder; see Figure 12 for its schematic structure. The encoder is a neural network that has the capability to encode the input *X* into latent features that contain compact and discriminative representatives *z*. The decoder is also a neural network that has the ability to decode these representative features into their original form $X^{\prime }$. The network is trained to reduce the error value *e* computed by the cost function Θ between *X* and $X^{\prime }$.

**Figure 13:**
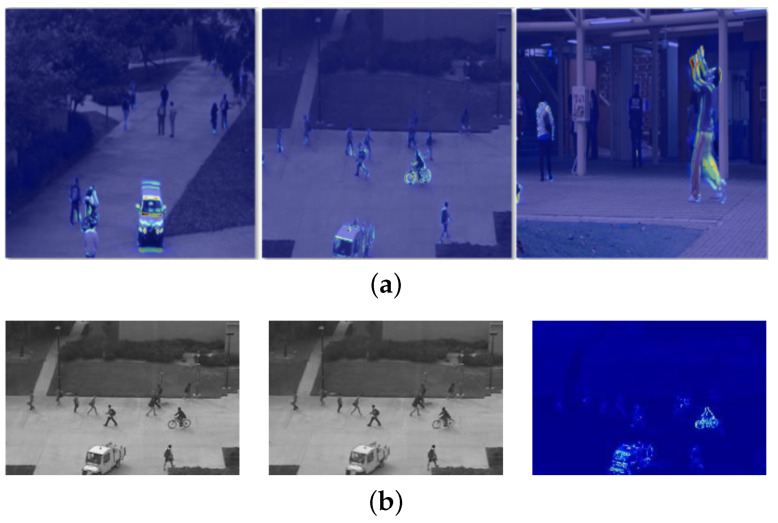

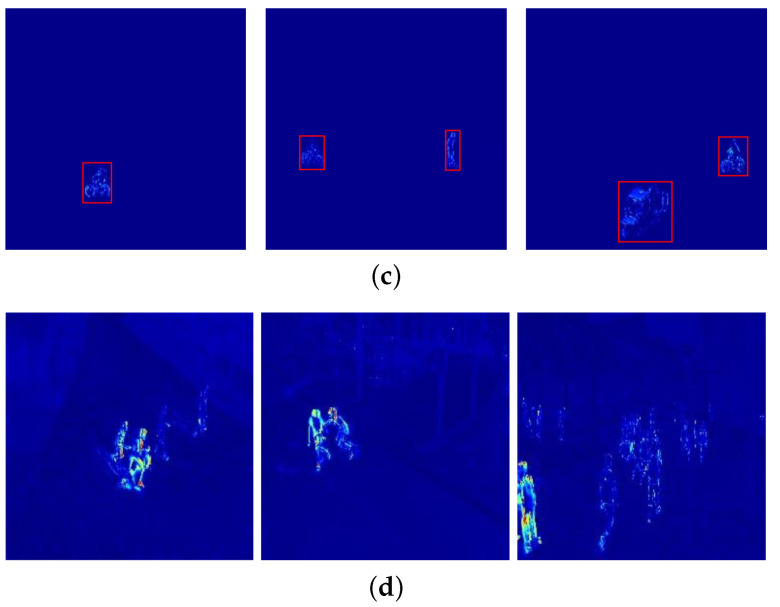
Error maps from recent methods. (**a**) Zero-shot cross-domain video anomaly detection (zxVAD)—Aich et al. [100] (2023); (**b**) dynamic local aggregation network—Yang et al. [101] (2022); (**c**) spatialtemporal memories—Liu et al. [102] (2022); (**d**) hybrid attention and motion constraint—Zhang et al. [103] (2022).

Many approaches have utilized an autoencoder structure for identifying anomalies. The work in [81] used a sparse coding autoencoder to preserve the spatiotemporal information between the input and output. They used a 2D convolution network to encode the grayscale 2D image sequences from each segment of the videos. The authors only used one-channel input image stacking in the temporal dimension, for better reconstruction in both the spatial and temporal domains. The work of [4] also used a reconstruction-based method as their approach to AbHAR. However, they enhanced the spatiotemporal information using a convolutional long-short-term memory (Conv-LSTM) network as their main architecture. A LSTM network [99] has the ability to capture the long-term information of video data and hence increase the prediction accuracy. The researchers in [86] used low-level features such as edges and optical flow to add extra information to raw frames. Another use of autoencoders was proposed in [82], where the authors used two different autoencoders for their task: the first was a normal autoencoder, and the second was a sparse autoencoder that constrained the dimensional features and retained the most useful active neurons in the latent layer (see Figure 13).

In [84], the authors used a 3D convolution autoencoder to preserve the temporal information and keep track of the spatial features in the temporal dimension. They also applied data augmentation, to increase the number of training samples. Later, the authors in [89] proposed a novel network for a feature learning framework that combined both motion and appearance features in an image, namely SC2NET. This network had the capability to compute sparsity loss and was trained through construction error, to learn how to construct useful spatiotemporal features.

The approach using an autoencoder is based on the assumption that a network will return a high construction error score for an abnormal instance. However, this assumption does not necessarily hold true, since, in some cases, autoencoders can generalize the abnormal instance as well as the normal instance. This means the reconstruction error score is lower than expected. The researchers in [92] proposed new approach to deal with this problem. They treated each encoding feature as a query for the decoder network. All normal encoding features were stored into memory. The decoder returned the closest normal encoding in the memory to each query instance. An abnormal instance hence cannot map the closest normal encoding and return a high reconstruction error (see Table 2).

### 5.2. Multiclass Classifier Method

Despite the popularity of using error reconstruction as the main method for anomaly detection. Different approaches have framed the anomaly detection problem as a multiclass classification. This classification method receives the video segment as an input *x* and returns an output *y* indicating a class label in redefined categories, see Equation (2).

(2) y=f(x),y∈R

One of the main problems in anomaly detection is imbalanced datasets, and the approach in [3] tried to resolve this problem using a cascade framework to learn compact and robust features. There are two stages in this approach, the first uses a stack of autoencoders to only learn from the normal video patches. The second stage is a convolutional neural network, which processes the video patches that cannot be modeled in the first stage and need further investigation. The final features are put into a Gaussian classifier to perform classification. Another approach seen in [83] used both local and global descriptors to learn the representative features from a video segment. These features were composed of both spatial and temporal information from each video patch. For a local descriptor, they used a similarity metric to extract spatiotemporal features. Meanwhile, the global descriptor utilized a pretrained autoencoder network to learn latent features. Next, these features were put into another autoencoder to select the most representative ones and the selected features were fed into a Gaussian classifier to detect anomalies. The authors in [85] also used Gaussian classifiers to make a prediction; however, they took advantage of pretrained models and extracted intermediate features, to find robust patterns for the classifiers to detect anomalies. In cases where the classifier could not make a decision, the features were sent to the top layers in the pretrained models, to learn more discriminative features.

The work of [93] also used pretrained models to learn representative features. They explored the feature spaces of a convolutional neural network in different domains, using the technique of transfer component analysis [118] to make a generalization. The method proposed in [94] focused on each human object in a scene using a single-shot detector model (SSD) [119] for each frame in the video. After locating each target subject in the scene, two different autoencoder models were used to learn both motion and appearance features for each subject. These features were concatenated and clustered into different subsets using a k-mean technique [120]. Each cluster represented one kind of normal event. A one-versus-rest classifier was trained to perform classification of each cluster. Any event that received a negative score from the classifier and did not belong to any cluster was tagged as an abnormal event. Ref. [96] also framed the anomaly detection problem as a multiclass classification problem, by combing all the representative features from the optical flow and gradient in each video patch. This allowed the author to build an adaptive intraframe classification network that learned both motion and appearance features, to detect and localize anomalies in video patches.

### 5.3. Future Frame Prediction Method

One problem with reconstruction-based methods is that the autoencoder can accidentally reconstruct abnormal instances as well as normal ones. This may produce many false negatives and reduce the performance. The work of [29] suggested a new method, named future frame prediction, that can handle this problem well. Formally, given $x_{t}$ is the input video segment at time step *t*, the future frame prediction method provides a function *p* to predict the next segment frames at time t+1 and compare the error cost between the predicted frames and the current frames at that time. If the error value is greater than a defined threshold value, then the frames are tagged as abnormal instances. Equation (3) gives the mathematical form for this approach.

(3) $$x_{t+1}=p\left(x_{t}\right)$$

A neural network called generative adversarial networks (GANs) [43] is used for this approach. Basically, GANs is a generative model that comprises two main parts: a generator, and a discriminator. The generator is used for generating new instance data based on statistical information from the training data. The discriminator’s job is to verify whether the input is coming the generator (fake) or coming from training data (real). Ref. [121] used GANs in their approach. The generator was a neural network using a U-Net architecture [122] to predict future frames. The authors chose the U-Net model since it has shown significant performance in image-to-image translation tasks. The discriminator was a neural network used to determine the frames having abnormal events or not.

Some previous works on reconstruction-based methods also took advantage of this future frame prediction approach. For example, the approach in [81] encoded both spatial and temporal information from video segments and further predicted the future frame given a center frame. Some earlier works, such as ref. [84] and ref. [4], also leveraged the idea of future frame prediction to reconstruct the current frame. Reference [84] used two separated branches to learn how to predict the future frames and reconstruct the frame at the same time. The author used the loss value to train the network to extract temporal information. The loss value was a combination of both the prediction loss and the reconstruction loss. The approach in [4] also used a parallel branch to learn both frame prediction and reconstruction. However, their network could identify the point of interest within a video, to extract the useful information for detecting anomalies.

### 5.4. Scoring Method

In the scoring method, the network tries to predict the anomaly score for each video segment. Thus, it can be considered a regression problem, where the purpose is to assign a high score value (or sometimes a low score value) for any abnormal instance. Mathematically, the scoring method uses a function *s* to take the input video segments *x* and assign a *t* value indicating the anomaly score in each segment, see Equation (4).

(4) t=s(x),t∈R

The approach in [9] used a sum-squared derivative to calculate the anomaly score from the extracted features. These features comprised both deep and slow feature analysis [123], which was used to learn the semantic meaning from image sequences. The work in [2] used the technique of multiple-instance learning to measure the anomaly score from weakly labeled video segments. They utilized a 3D convolutional neural network [124] to learn both spatial and temporal information from the image sequences. The spatiotemporal features were fed into the network model. The model was trained with a supervised method, with weak labels to detect the final score for each video segment. The idea of using multiple-instance learning inspired the work in [90], where they used an optical flow feature along with an attention mechanism [125] to determine the promising features in video segments, to calculate the final score. Similarly based on an idea in [2], reference [91] proposed a new dual network to learn motion features by modifying the original model structure. The authors added an attention module and utilized social fore maps [39] to learn motion representatives.

The authors in [87] used optical flow to foreground objects from the background video. Then a convolutional neural network was used to extract the identified objects’ features, along with a histogram of optical flow. All the features were combined using a matrix factorization method and clustered into different subsets. A voting system detected whether new instances were normal or abnormal. The approach in [88] focused on localization in video segments, by extracting a tube from the video. They also showed that their method could accurately identify anomalies based on scoring values.

The researchers in [95] used sparse coding for anomaly detection. The sparse coding [41] first learned the dictionary mapping from all normal instances. The authors in [95] proposed temporal-coherent sparse coding using both spatial and temporal information extracted from a stacked recurrent neural network autoencoder. The work in [97] used a Gaussian mixture model for anomaly detection. They proposed a new network, named Gaussian mixture fully convolutional variational autoencoder, to model video patches using both motion and appearance features. The model predicted the probability score for each video patch through a simple energy-based method.

### 5.5. Anomaly Score

The difference between the predicted frame/reconstructed frame $\hat{I}$ and the ground truth frame *I* is computed using the peak signal-to-noise ratio (PSNR):

(5) $$\mathrm{PSNR}\left(I,\hat{I}\right)=10log_{10}\frac{{\left[max_{\hat{I}}\right]}^{2}}{\frac{1}{N}\sum_{i=1}^{N}{\left(I_{i}-{\hat{I}}_{i}\right)}^{2}}$$

The PSNR scores of all frames in a video are normalized to the range [0, 1]. Then, the anomaly score is obtained using the following formula:

(6) $$S\left(t\right)=\frac{{\mathrm{PSNR}}_{t}-min\left(\mathrm{PSNR}\right)}{max(\mathrm{PSNR})-min(\mathrm{PSNR})}$$

The anomaly score S(t) is used to determine whether a frame is a normal or abnormal event.

## 6. Research Gaps, Challenges, and Future Research

### 6.1. Research Gaps

Recent methods [43,126,127] have focused on exploiting both the appearance and motion information in video, by extracting structural features and the optical flow. The extraction of optical flow requires a high computational cost. Therefore, some proposed methods [116,128,129] have tried to capture motion information without using optical flow.

On the other hand, most recently proposed methods have tried to improve the accuracy of anomaly detection systems by applying a modern model [130,131]. We need to consider the complexity of the model, since an anomaly detection system should be run as a real-time application.

### 6.2. Challenges

This section discusses the general challenges in AbHAR for video surveillance, as well as possible solutions and future research. Overall, researchers have relied on supervised learning methods for their approach. This requires a large amount of labeled data for training and improving the model accuracy. However, collecting data is a tedious and laborious process. There is also no standard dataset that can cover all abnormal behaviors in real situations. These problems can be solved by utilizing transfer learning techniques or crowd-sourcing [132]. In addition, there is a need for standard metrics that allow a fair comparison between different approaches [133].

During inference, there are also some limitations in most approaches, such as false alarms due to subtle details in human motion and appearance. In some cases, early detection is also necessary for crime prevention. These challenges can be approached by choosing representative features for AbHAR [134] that allow the surveillance system to learn and simulate human behaviors. Intraclass variation and interclass similarity problems occur when a model considers nonsimilar patterns between the same activity or similar representatives among different classes. This can lead to an ambiguous boundary between the normal and abnormal events, and requires the model to learn only unique and discriminative features for classification [135]. Moreover, the tracking of multisubject interactions in a group of people is a challenging problem that requires a model to have the ability to capture spatiotemporal information from subjects [136].

A noisy environment is considered the main issue in many surveillance systems. A cluttered and dynamic background is challenging to model accurately. In addition, occlusion, low illumination, low-quality videos, and various viewpoints also occur in real-time surveillance. Multimodal data can overcome these challenges [137,138], e.g., the usage of D-RGB images to extract depth information from a video segment. Moreover, a surveillance system requires real-time sensing, which is highly energy consuming and requires a lot of computing power. This raises the requirement of using adaptive methods based on the principle of a sampling frequency and iterative segmentation [139].

### 6.3. Future Research

Future research will focus on using transfer learning methods in deep learning approaches, [140] such as pretrained models to explore the spatiotemporal relationships in video segments. In the context of AbHAR, transfer learning can be used to transfer the knowledge gained from a large-scale dataset to a small-scale dataset, where the latter may have different distributions and challenges. Future research could investigate the effectiveness of transfer learning in AbHAR using deep learning-based methods. Researchers currently pay more attention of how to interpret a video model by selecting key frame information [64,77] to classify activity classes. Moreover, extracting rich semantic features from multimodal datasets is a research trend [75,141], since this information enables explaining the long-term relationship between interacting objects [142]. Lastly, the physical interactions between humans or humans–objects [143] require more investigation, since these interactions can provide useful features for understanding human behaviors and provide more interpretive features for model training.

## 7. Conclusions

Video surveillance systems are becoming increasingly important in detecting and preventing criminal activities in various settings, such as public spaces, offices, and homes. One of the essential components of a video surveillance system is abnormal human activity recognition (AbHAR). AbHAR is critical for detecting and understanding anomalous behaviors, which could be potential threats to the safety and security of the environment being monitored. This paper provides a comprehensive review of deep learning-based methods in video surveillance. Human activity recognition plays an important role in detecting and understanding anomalous behaviors. This review has covered the essential aspects of related works and background knowledge. It has also presented different benchmark databases used in AbHAR, with a detailed description of each one. These databases are increasing in both quantity and quality, allowing researchers to study many aspects related to real-time scenarios. This comprehensive review of deep learning-based methods has introduced methods such as reconstruction based approaches, multiclass classification, future frame prediction, and scoring methods. This diversity of approaches demonstrates the difficulty of AbHAR, which has forced researchers to find better solutions. In addition, future studies on the transfer learning method or multimodal datasets will provide initial steps toward a better analysis in anomaly detection. In fact, the choice of representative features in the feature engineering step is the most difficult part in any surveillance system. Robust feature extraction from a dynamic background is an essential part of capturing relative and discriminative patterns for tracking and understanding human behaviors.

## Figures and Tables

**Table 1:** Summary of deep learning-based techniques for anomaly detection.

Method	Type	Year	Reference
Convolutional Long Short-Term Memory	RB + Future Frame Prediction	2016	[4]
2D Convolutional Autoencoder	RB	2016	[81]
Sparse Autoencoder	Reconstruction based	2016	[82]
Slow Feature Analysis + Deep Neural Network	Scoring	2016	[9]
Sparse Denoise Autoencoder	Multiclass Classification	2017	[83]
Autoencoder + Cascade Deep CNN	Multiclass Classification	2017	[3]
Spatiotemporal Autoencoder	RB + Future Frame Prediction	2017	[84]
Pretrained DNN + Gaussian classifier	Multiclass Classification	2018	[85]
Autoencoder + Low level features	Reconstruction based	2018	[86]
Multiple Instance Learning	Scoring	2018	[2]
Low-level Features + Autoencoder	Reconstruction based	2018	[86]
Frame predict using GANs	Future Frame Prediction	2018	[43]
Combination of traditional and deep features	Scoring	2019	[87]
Localization feature extraction	Scoring	2019	[88]
AnomalyNet	Reconstruction based	2019	[89]
Optical Flow + Multiple Instance Learning	Scoring	2019	[90]
Social Force Maps + Multiple Instance Learning	Scoring	2019	[91]
Attention module + Autoencoder	Reconstruction based	2019	[92]
Component Analysis + Transfer Learning	Multiclass Classification	2019	[93]
Object detection using SSD + Autoencoder	Multiclass Classification	2019	[94]
Sparse coding Deep neural network	Scoring	2019	[95]
Adaptive Intra-Frame Classification Network	Classification	2019	[96]
Autoencoder + Gaussian Mixture Model	Scoring	2020	[97]

**Table 2:** Comparison of recent methods used for video anomaly detection.

Method	Ped 1	Ped 2	Avenue	ShanghaiTech	Year	Reference
Cognitive memory-augmented network (CMAN)	-	96.2	-	-	2021	[104]
Single and multi-frame anomaly detection	-	97.5	87.2	-	2021	[105]
Multi-Level Memory modules in an Autoencoder with Skip Connections (ML-MemAE-SC)	-	99.3	91.1	76.2	2021	[106]
Autoencoder with a Memory Module (AMM)	-	97.2	87.9	70.2	2021	[107]
Explanation for Anomaly Detection	73.1	80.1	-	-	2021	[108]
Attention-based adversarial autoencoder (A3N)	90.7	97.7	89.4	86.9	2022	[109]
Group Activities for AD	84.4	95.0	82.3	-	2022	[110]
Variational Anomaly Detection Network (VADNet)	-	96.8	87.3	75.2	2022	[111]
Context-related video anomaly detection	-	96.3	87.1	73.6	2022	[112]
Localization based Reconstruction (LBR)	81.1	97.2	92.8	72.6	2022	[113]
Foreground–Background Separation Mutual Generative Adversarial Network (FSM-GAN)	-	98.1	80.1	73.5	2022	[114]
Dual-stream memory network	-	98.3	88.6	75.7	2023	[115]
Attention-based residual autoencoder	-	97.4	86.7	73.6	2023	[116]
Bi-directional Frame Interpolation	-	98.9	89.7	75.0	2023	[117]
Zero-shot Cross-domain Video Anomaly Detection (zxVAD)	78.6	95.8	83.2	-	2023	[100]

## Data Availability

The datasets generated during and/or analyzed during the current study are available from the corresponding author on reasonable request.
